# Aurora kinase targeting in lung cancer reduces KRAS-induced transformation

**DOI:** 10.1186/s12943-016-0494-6

**Published:** 2016-02-03

**Authors:** Edmilson Ozorio dos Santos, Tatiana Correa Carneiro-Lobo, Mateus Nobrega Aoki, Elena Levantini, Daniela Sanchez Bassères

**Affiliations:** Department of Biochemistry, Chemistry Institute, University of São Paulo, São Paulo, SP Brazil; Beth Israel Deaconess Medical Center, Harvard Medical School, Boston, MA USA; Institute of Biomedical Technologies, National Research Council (CNR), Pisa, Italy

## Abstract

**Background:**

Activating mutations in KRAS are prevalent in lung cancer and have been causally linked to the oncogenic process. However, therapies targeted to oncogenic RAS have been ineffective to date and identification of KRAS targets that impinge on the oncogenic phenotype is warranted. Based on published studies showing that mitotic kinases Aurora A (AURKA) and B (AURKB) cooperate with oncogenic RAS to promote malignant transformation and that AURKA phosphorylates RAS effector pathway components, the aim of this study was to investigate whether AURKA and AURKB are KRAS targets in lung cancer and whether targeting these kinases might be therapeutically beneficial.

**Methods:**

In order to determine whether oncogenic KRAS induces Aurora kinase expression, we used qPCR and western blotting in three different lung cell-based models of gain- or loss-of-function of KRAS. In order to determine the functional role of these kinases in KRAS-induced transformation, we generated KRAS-positive A549 and H358 cells with stable and inducible shRNA-mediated knockdown of AURKA or AURKB and evaluated transformation in vitro and tumor growth in vivo. In order to validate AURKA and/or AURKB as therapeutically relevant KRAS targets in lung cancer, we treated A549 and H358 cells, as well as two different lung cell based models of gain-of-function of KRAS with a dual Aurora kinase inhibitor and performed functional in vitro assays.

**Results:**

We determined that KRAS positively regulates AURKA and AURKB expression. Furthermore, in KRAS-positive H358 and A549 cell lines, inducible knockdown of AURKA or AURKB, as well as treatment with a dual AURKA/AURKB inhibitor, decreased growth, viability, proliferation, transformation, and induced apoptosis in vitro*.* In addition, inducible shRNA-mediated knockdown of AURKA in A549 cells decreased tumor growth in vivo. More importantly, dual pharmacological inhibiton of AURKA and AURKB reduced growth, viability, transformation, and induced apoptosis in vitro in an oncogenic KRAS-dependent manner, indicating that Aurora kinase inhibition therapy can specifically target KRAS-transformed cells.

**Conclusions:**

Our results support our hypothesis that Aurora kinases are important KRAS targets in lung cancer and suggest Aurora kinase inhibition as a novel approach for KRAS-induced lung cancer therapy.

**Electronic supplementary material:**

The online version of this article (doi:10.1186/s12943-016-0494-6) contains supplementary material, which is available to authorized users.

## Background

Activation of KRAS by mutation is a very common event in human malignancies. In spite of intensive investigation, KRAS-related malignancies currently lack effective therapies. Direct targeting of KRAS by blocking its post-translational prenylation has failed in clinical trials [1]. Targeting KRAS downstream effectors has also been challenging, as KRAS regulates a multitude of effectors that contribute to the oncogenic phenotype [2, 3]. It is likely that successful KRAS targeting will involve combined inhibition of specific key targets. Considering that targeting traditional KRAS effectors has so far had limited success [1, 4], the identification of novel KRAS targets that impinge on the oncogenic phenotype is warranted in order to increase the possibilities of combinatorial therapy design and achieve therapeutic efficacy.

Achieving therapeutic efficacy is particularly important in lung cancer, which is the leading cause of cancer-related deaths [5]. Even though effective targeted therapies have been developed for lung cancer, these therapies benefit a small percentage of patients because they target oncogenic events that are infrequent in lung cancer [6, 7]. KRAS mutations, however, are very common in lung cancer ranging from 30–50 % of patients and are associated with poor prognosis and therapy resistance [8, 9]. Nonetheless, effective targeted therapy options for lung cancer patients with KRAS mutations are currently lacking.

Aurora kinases A and B belong to a new family of serine/threonine kinases, which are essential regulators of mitosis [10, 11] and have been recently implicated in DNA repair [12, 13]. They are also overexpressed in a number of human cancers [14, 15], including lung cancers [16–19]. In addition, both kinases have been implicated in promoting oncogenesis [20–25]. Aurora A expression can transform cells and induce tumor formation in mice [24, 26] and Aurora B overexpression promotes lung carcinogenesis and increased invasiveness in vivo [25]. In addition, these kinases have been shown to promote genetic instability leading to aneuploidy [21, 26–29] and to block p53 function, thereby preventing cell apoptosis [30, 31]. Finally, these kinases have been shown to cooperate with RAS to induce malignant transformation [28, 32–37]. Even though these kinases are being investigated as therapeutic targets, and specific Aurora kinase inhibitors have been developed and are undergoing clinical trials for different malignancies [14, 15, 38], it is not known whether these kinases are KRAS targets in lung oncogenesis, or if targeting these kinases could lead to a therapeutic benefit for lung cancer patients harboring KRAS mutations.

In this study we investigated Aurora A and Aurora B as potential KRAS targets in lung cancer. We show, not only that, in lung cells, KRAS regulates Aurora A and B expression, but also that targeting these kinases in lung cells by different approaches reduces cell growth, proliferation and anchorage-independent growth, while at the same time it induces apoptosis. Interestingly, these effects were more pronounced in the presence of oncogenic KRAS^G12V^, and Aurora inhibition had no effect on normal or tumorigenic cells without KRAS mutations. This suggests that Aurora kinase inhibition therapy can specifically target KRAS transformed cells. Finally, AURKA inhibition by RNA interference reduced lung tumor xenograft growth in vivo. In conclusion, our results support our hypothesis that Aurora kinases are important KRAS targets in lung cancer and suggest Aurora kinase inhibition as a novel and specific approach to be explored for KRAS-induced lung cancer therapy.

## Results

### Oncogenic KRAS induces Aurora A and Aurora B expression

**Fig. 1:**
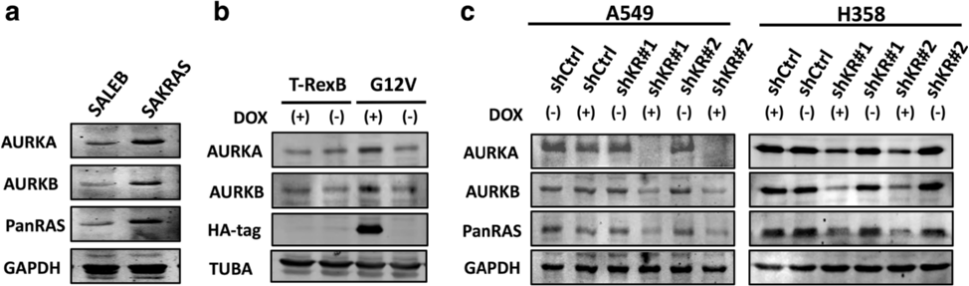
KRAS induces expression of AURKA and AURKB in lung cells. **a** Protein lysates of SALEB and SAKRAS cells were submitted to western blotting with the indicated antibodies. **b** H1703-TrexB and H1703-G12V lung cancer cells were treated with 2 μg/mL doxycycline (DOX) for 24 h where indicated to induce KRAS expression. Subsequently, protein lysates were prepared and submitted to western blotting with the indicated antibodies. TUBA) anti-α-tubulin. **c** A549 and H358 stable cells with inducible expression of 2 different shRNAs targeting KRAS (shKR#1 and shKR#2) or a non-targeting shRNA (shCtrl) were treated with 2 μg/mL doxycycline (DOX) for 5 days to induce shRNA expression where indicated. Protein lysates were prepared and submitted to western blotting with the indicated antibodies

Because both AURKA and AURKB can cooperate with RAS to promote malignant transformation [28, 32–37], and because AURKA can activate the RAS downstream effector RalA [39], we hypothesized that KRAS could promote Aurora kinase expression, as part of its oncogenic activity. In order to determine whether oncogenic KRAS regulates expression of AURKA or AURKB, we used three cell-based models. Initially, we used an isogenic pair consisting of immortalized primary pulmonary cells transformed or not with oncogenic KRAS [40]. KRAS-transformed SAKRAS cells express higher levels of RAS proteins in comparison with their non-transformed SALEB counterparts, as expected. Interestingly, SAKRAS cells also express higher levels of AURKA and AURKB (Fig. 1a). In order to determine if these increased Aurora kinase levels could reflect increased AURKA and AURKB transcription in SAKRAS cells, we performed RT-qPCR (Additional file 1: Methods) and found that, when compared to SALEB cells, SAKRAS cells have, not only a higher expression of KRAS mRNA, but also a higher expression of AURKA and AURKB mRNAs (Additional file 2: Figure S1A). Therefore, these results show a correlation between KRAS and Aurora kinase expression in primary pulmonary cells.

In order to determine if KRAS can induce Aurora kinase expression, we used both gain-of-function and loss-of-function cell based models. For gain-of-function of KRAS, we used HA-tagged KRAS^G12V^-inducible H1703 human lung cancer cells, that express oncogenic KRAS^G12V^ upon doxycycline administration [41]. We observed that induction of KRAS^G12V^ expression with doxycycline in H1703 cells, as assessed by immunoblotting with an anti-HA tag antibody, leads to increased AURKA and AURKB expression (Fig. 1b). These effects on Aurora kinase expression were not observed in doxycycline-treated empty vector control cells (H1703-TrexB cells), which do not express KRAS^G12V^ (Fig. 1b). Similarly to what we observed in SAKRAS cells, induction of KRAS^G12V^ expression in H1703 cells leads to increased AURKA and AURKB mRNA levels (Additional file 2: Figure S1B).

Next, we performed loss-of-function experiments using KRAS mutant H358 and A549 cell lines with doxycycline-inducible expression of shRNAs targeting KRAS. shRNA expression upon doxycycline administration was monitored by fluorescence microscopy for the red fluorescent reporter protein (Additional file 2: Figure S1C). Consistent with our previous results, inhibition of KRAS expression with two different shRNAs in both A549 and H358 cell lines is accompanied by a decrease in AURKA and AURKB expression (Fig. 1c). Again, KRAS, AURKA and AURKB expression is unchanged in A549 and H358 cells induced to express a non-targeting shRNA (Fig. 1c). Finally, this decrease in AURKA and AURKB protein levels is accompanied by a decrease in mRNA levels (Additional file 2: Figure S1D). Taken together, these results confirm that, in lung cells, AURKA and AURKB are KRAS targets, and strongly suggest that KRAS signaling upregulates AURKA and AURKB transcription or promotes AURKA and AURKB mRNA stability.

### Targeting Aurora A and Aurora B reduces the oncogenic phenotype of KRAS-positive lung cancer cells

The above results, coupled to the fact the both AURKA and AURKB have been implicated in promoting the malignant phenotype [20–25], led us to hypothesize that AURKA and AURKB contribute to the oncogenic phenotype induced by KRAS. In order to test this hypothesis, we assessed if simultaneous inhibition of AURKA and AURKB activity with Aurora Kinase Inhibitor II (AI II), a dual Aurora kinase pharmacological inhibitor, would affect KRAS-positive lung cell oncogenicity.

**Fig. 2:**
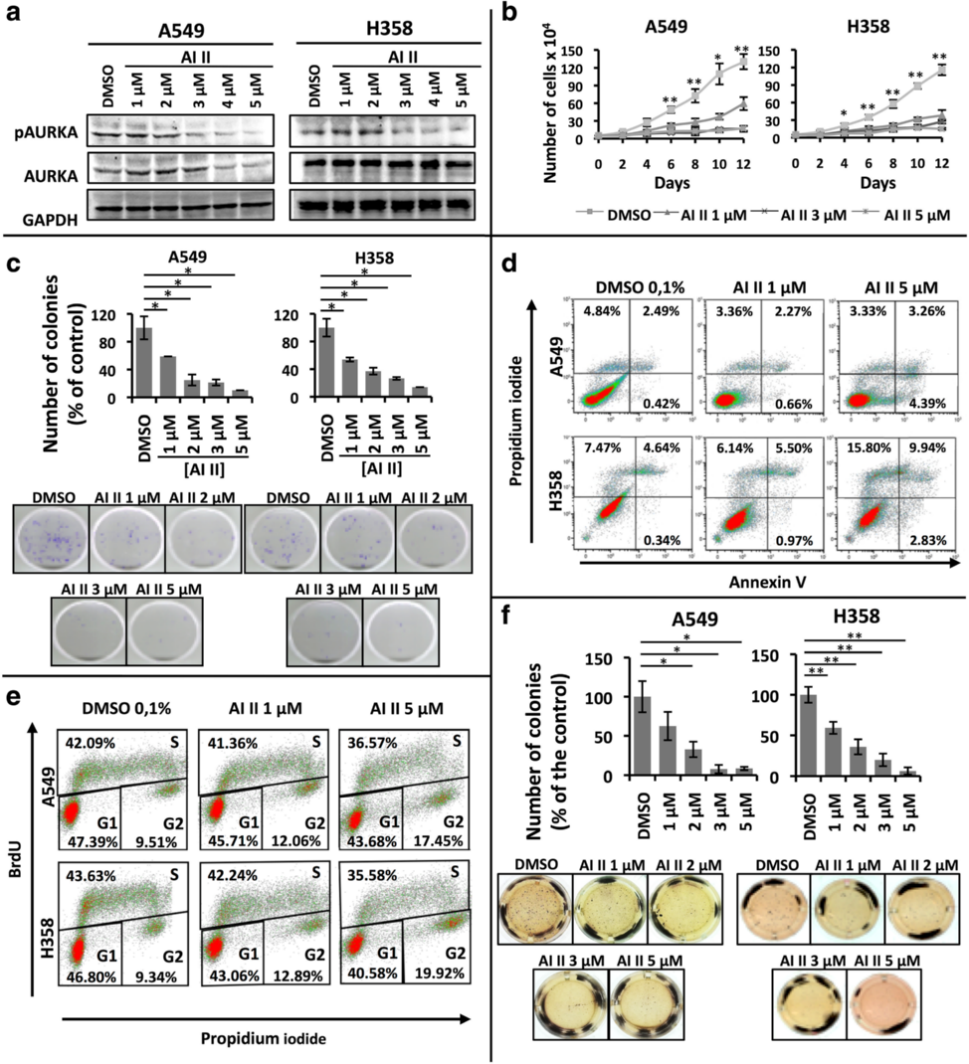
Dual pharmacological inhibition of AURKA and AURKB decreases the transformed phenotype of KRAS-positive lung cells. **a** A549 and H358 cells were treated with 0.1 % DMSO or increasing concentrations of AI II as indicated for 72 h and protein lysates were prepared and submitted to Western blotting with the indicated antibodies. **b** Growth curve analysis of A549 and H358 cells treated with the indicated concentrations of AI II compared to control-treated cells (0.1 % DMSO) for the indicated times. **c** A549 and H358 cells were plated for clonogenic assays as described in methods and treated for 21 days with either 0.1 % DMSO or different concentrations of AI II as indicated. Colonies formed were stained with crystal violet and counted. Images shown are representative of three independent experiments. **d** A549 and H358 cells were treated with 0.1 % DMSO or increasing concentrations of AI II as indicated for 72 h, stained for Annexin V and propidium iodide (PI) as described in methods and Annexin V positive cells were analyzed by flow cytometry. **e** A549 and H358 cells were treated with 0.1 % DMSO or increasing concentrations of AI II as indicated for 72 h, cells were stained with BrdU and PI as described in methods, and cell cycle analysis was performed by flow cytometry. **f** Anchorage-independent growth was evaluated by plating A549 and H358 in soft agar as described in methods. Cells were then treated for 21 days with either 0.1 % DMSO or different concentrations of AI II as indicated. Colonies formed were stained with MTT and counted. Images shown are representative of three independent experiments. In all cases, statistical significance was determined when appropriate by Student’s *t*-test (*p < 0.05, **p < 0.01) by comparing AI II-treated vs. DMSO-treated samples. Error bars represent average ± 1 s.d

Because AI II has higher affinity for AURKB [42, 43], we decided to investigate first if AI II can effectively inhibit AURKA activity under the conditions used in our experiments in order to validate dual inhibition of these kinases. AURKA activity was assessed by the level of autophosphorylation at threonine 288, which is required for AURKA activity [44, 45]. As can be seen in Fig. 2a, AI II inhibits AURKA autophosphorylation in a dose-dependent manner.

Treatment of KRAS-positive cancer cell lines with AI II effectively reduces, in a dose-dependent manner, cell growth (Fig. 2b), as well as the ability to form colonies under adherent conditions (Fig. 2c). This AI II-induced loss of viability was corroborated by MTT reduction-based assays, which show a similar dose-dependent reduction of cell viability (Additional file 3: Figure S2A).

In both cell lines, AI II treatment resulted in an accumulation of annexin V positive cells (Fig. 2d) and induced caspase 3 cleavage (Additional file 3: Figure S2B). In addition, AI II caused a reduction in the number of cells in S phase and caused an accumulation of cells in the G2 phase of the cell cycle (Fig. 2e). These results indicate that Aurora kinase inhibition in KRAS-positive cells not only reduces cell proliferation, but also promotes apoptosis.

One important cell property that is associated with the transformed phenotype is the ability of cells to grow in an anchorage-independent manner. As can be seen in Fig. 2f, AI II reduces, in a dose-dependent manner, the ability of both cell lines to form colonies under non-adherent conditions.

**Fig. 3:**
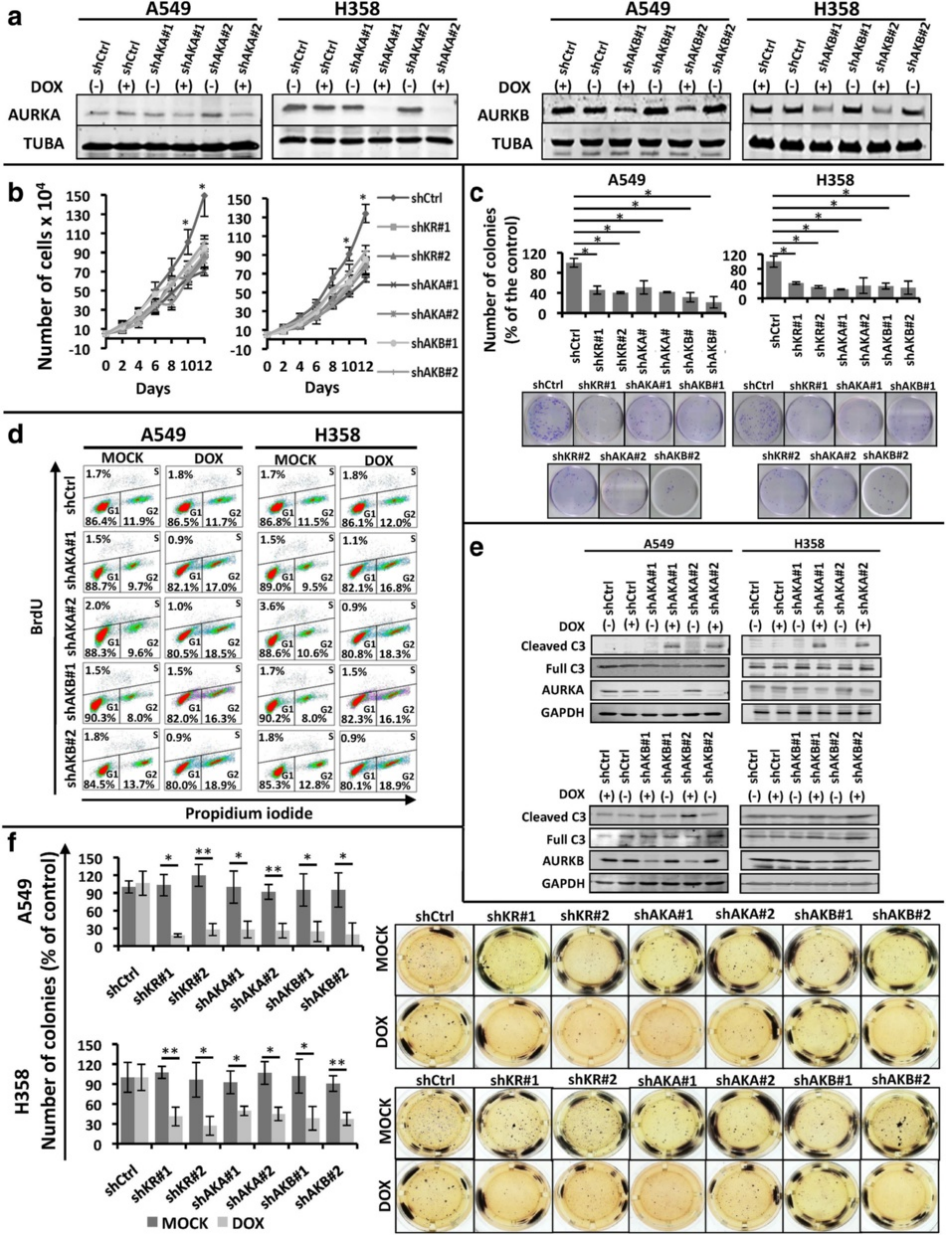
shRNA-mediated knockdown of AURKA or AURKB decreases the transformed phenotype of KRAS-positive lung cells. Unless otherwise indicated, A549 and H358 stable cells with inducible expression of 2 different shRNAs targeting AURKA (shAKA#1 and shAKA#2), AURKB (shAKB#1 and shAKB#2) or a non-targeting shRNA (shCtrl) were either treated with 2 μg/mL doxycycline (DOX) for 5 days to induce shRNA expression or left untreated (MOCK). **a** Protein lysates of doxycycline-treated (+) and untreated (−) cells were submitted to western blotting with the indicated antibodies. TUBA) anti-α-tubulin. **b** Growth curve analysis of the indicated cells. All cells were treated with 2 μg/mL doxycycline (DOX) for the indicated times. **c** The indicated cells were plated for clonogenic assays as described in methods and treated for 21 days with 2 μg/mL doxycycline (DOX). Colonies formed were stained with crystal violet and counted. Images shown are representative of three independent experiments. **d** The indicated treated (DOX) or untreated (MOCK) cells were stained with BrdU and propidium iodide (PI) as described in methods, and cell cycle analysis was performed by flow cytometry. **e** Protein lysates of A549 and H358 stable cells with inducible expression of 2 different shRNAs targeting AURKA (shAKA#1 and #2), AURKB (shAKB#1 and #2) or a non-targeting shRNA (shCtrl), treated (+) or not (-) with 2 μg/mL doxycycline (DOX) for 5 days, were submitted to western blotting with the indicated antibodies. C3) anti-caspase 3. **f** Anchorage-independent growth was evaluated by plating the indicated cells in soft agar as described in methods. Cells were then treated for 21 days with 2 μg/mL doxycycline (DOX) or left untreated (MOCK). Colonies formed were stained with MTT and counted. Images shown are representative of three independent experiments. In all cases, statistical significance was determined when appropriate by Student’s *t*-test (*p < 0.05, **p < 0.01) and the groups being compared are indicated by horizontal bars

In order to determine the role of each individual Aurora kinase in promoting KRAS-induced oncogenesis, we performed loss-of-function experiments using KRAS mutant H358 and A549 cell lines with doxycycline-inducible expression of shRNAs targeting AURKA or AURKB independently (Additional file 2: Figure S1C and D). Consistent with our previous results, doxycycline administration in both cell lines leads, not only to effective inhibition of AURKA or AURKB expression with two different shRNAs each (Fig. 3a), but also to reduced growth (Fig. 3b) and reduced ability to form colonies under adherent conditions (Fig. 3c). Again, loss of viability was confirmed by MTT reduction-based assays (Additional file 3: Figure S2C). As expected, these effects are not seen in A549 and H358 cells induced to express a non-targeting shRNA control upon doxycycline administration.

Similar to what we observed with AI II treatment, shRNA-mediated knockdown of AURKA and AURKB was accompanied by a reduction of the ability of these cells to progress through the cell cycle, leading to accumulation at G2 (Fig. 3d), as well as increased levels of cleaved caspase 3 (Fig. 3e). Finally, AURKA or AURKB knockdown in these cells also reduced anchorage-independent growth (Fig. 3f).

These results indicate that AURKA and AURKB inhibition reduces proliferation and promotes apoptosis, thereby leading to reduced growth and viability, as well as reduces the ability of KRAS-positive lung cancer cells to maintain a transformed phenotype.

### shRNA-mediated inhibition of Aurora A expression reduces KRAS-induced tumor growth in vivo

In order to validate Aurora kinases as potential therapeutic targets for KRAS-induced lung cancer, it is important to demonstrate that Aurora kinase targeting reduces KRAS-driven tumor growth in vivo. Given that the results we obtained in vitro were similar for both AURKA and AURKB targeting, and given that AURKA has been more extensively implicated in promoting oncogenesis, we performed in vivo studies with KRAS mutant A549 cells with inducible inhibition of AURKA expression to evaluate the effect of inhibition of AURKA expression for KRAS-positive lung tumor xenograft growth.

**Fig. 4:**
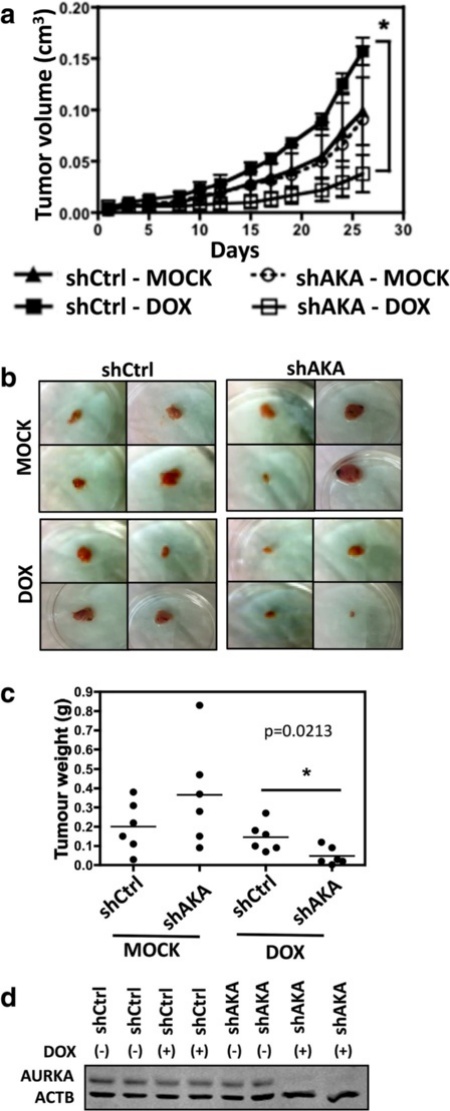
shRNA-mediated knockdown of AURKA reduces xenograft tumor growth. **a** A549 stable cells with inducible expression of a shRNA targeting AURKA (shAKA) or a non-targeting shRNA (shCtrl) were injected subcutaneously in nude mice (*n* = 9 per group). shRNA expression was induced by doxycycline (DOX) administration in the drinking water as described in methods (*n* = 5 per group). Alternatively, mice were left untreated (MOCK) to control for doxycycline-mediated effects (*n* = 4 per group). The graph shows tumor volume measurements, which were initiated 26 days after inoculation (day 0). Error bars represent average ± 1 s.d. **b** Representative images of the tumors at day 56 after inoculation. **c** A549 stable cells with inducible expression of a shRNA targeting AURKA (shAKA) or a non-targeting shRNA (shCtrl) were injected subcutaneously in nude mice (*n* = 12 per group). shRNA expression was induced by doxycycline (DOX) administration in the drinking water as described in methods (*n* = 6 per group). Tumor weights at day 56. Error bars represent average ± 1 s.d. In all cases, statistical significance was determined when appropriate by one-way analysis of variance (ANOVA) followed by Bonferroni’s multiple comparison test (**a**) or by Student's *t*-test (**c**) (*p < 0.05) and the significantly different comparisons are indicated by vertical (**a**) or horizontal (**c**) bars. **d** Protein lysates of xenograft tumors were submitted to western blotting with the indicated antibodies. ACTB) anti-β-actin

For that purpose A549 cells with doxycycline-inducible expression of a shRNA targeting AURKA (A549-shAKA) or with doxycycline-inducible expression of a non-targeting shRNA (A549-shCtrl) were inoculated subcutaneously in nude mice. Doxycycline administration in mice inoculated with A549-shAKA cells significantly reduced tumor growth rate when compared to untreated mice inoculated with these cells, as well as to doxycycline-treated and untreated mice inoculated with A549-shCtrl cells (Fig. 4a). Consistently, doxycycline-treated A549-shAKA tumors were smaller (Fig. 4b), displayed reduced weight (Fig. 4c) and reduced AURKA expression (Fig. d). Nonetheless, AURKA inhibition did not lead to tumor regression (Fig. 4a). This is consistent with the fact that apoptotic cells were not detected in these tumors (data not shown).

### Inhibition of Aurora kinase activity reduces the oncogenic properties of lung cells in a KRAS^G12V^-dependent manner

In order to address if Aurora inhibition preferentially affects KRAS-transformed cells, we used our previously described cell-based models to assess the effects of pharmacological inhibition of Aurora kinase activity in the presence and absence of oncogenic KRAS expression.

**Fig. 5:**
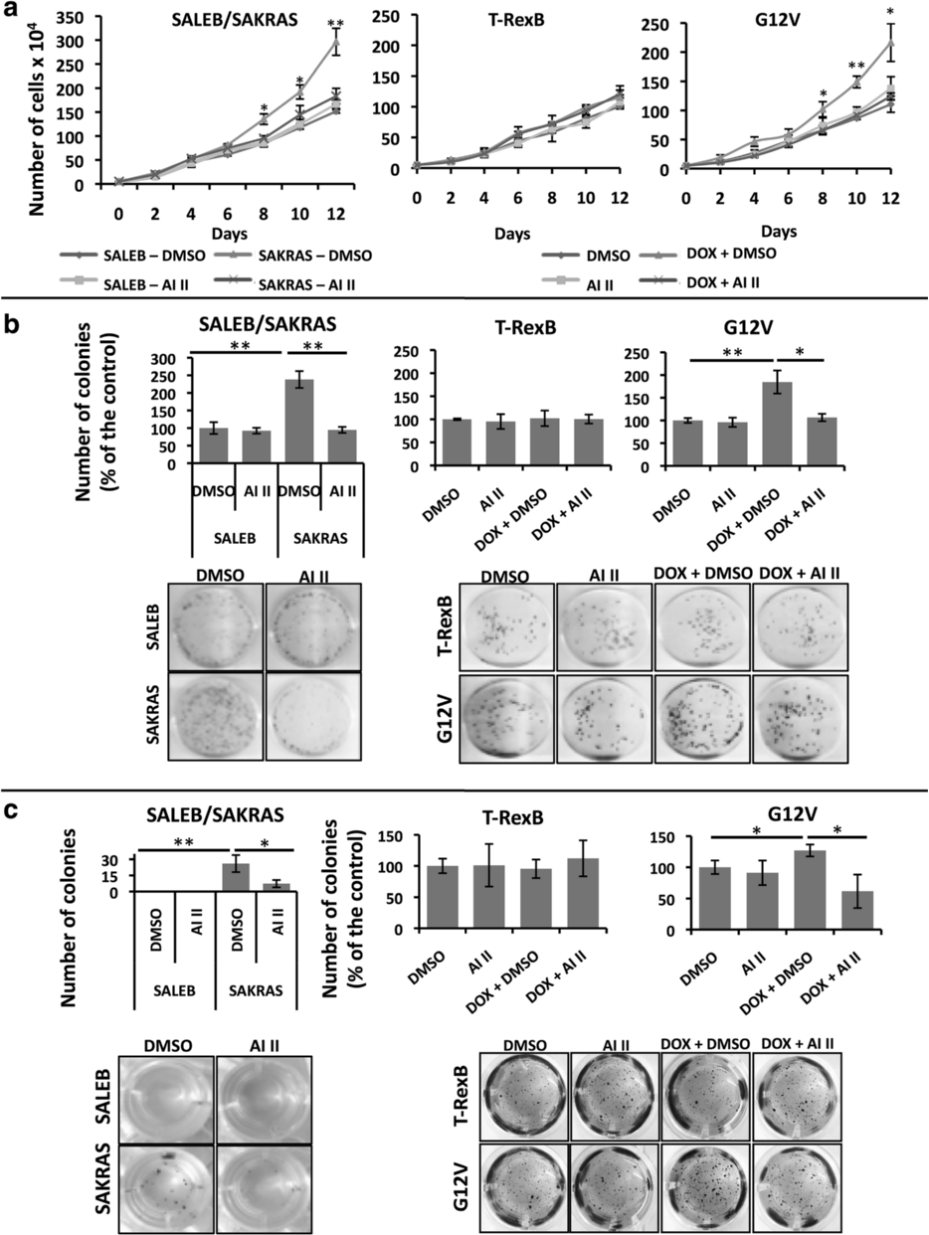
AURKA or AURKB targeting decreases the transformed phenotype of lung cells in a KRAS-dependent manner. Primary immortalized human airway cells (SALEB) and their KRAS-transformed counterpart (SAKRAS) were treated with 0.1 % DMSO (DMSO) or 1uM AI II (AI II) as indicated. H1703-TrexB and H1703-G12V lung cancer cells were also treated with 0.1 % DMSO (DMSO) or 1 μM AI II (AI II) as indicated. To induce KRAS expression H1703-TrexB and H1703-G12V cells were simultaneously treated with 2 μg/mL doxycycline (DOX + DMSO or DOX + AI II) where indicated. **a** Growth curve analysis of cells. All drug treatments (DMSO, AI II, DOX + DMSO, DOX + AI II) were continued for 12 days. **b** Cells were plated for clonogenic assays as described in methods and treated for 21 days as indicated. Colonies formed were stained with crystal violet and counted. Images shown are representative of three independent experiments. **c** Anchorage-independent growth was evaluated by plating the indicated cells in soft agar as described in methods. Cells were then treated for 21 days as indicated. Colonies formed were stained with MTT and counted. Images shown are representative of three independent experiments. In all cases, statistical significance was determined when appropriate by Student’s *t*-test (*p < 0.05, **p < 0.01) and the groups being compared are indicated by horizontal bars

As expected, SAKRAS cells grow faster than SALEB cells (Fig. 5a, left panel) and form more colonies under adherent conditions (Fig. 5b, left panel). Interestingly, treatment with 1uM AI II reduces growth and colony formation of SAKRAS cells without affecting the ability of untransformed SALEB cells to grow (Fig. 5a, left panel) and form colonies (Fig. 5b, left panel). In addition, only SAKRAS cells are able to form colonies in soft agar, a feature of transformed cells, and this ability is reduced by AI II treatment (Fig. 5c, left panel).

Similarly, induction of KRAS^G12V^ expression by doxycycline in H1703-G12V cells leads to enhanced growth (Fig. 5a, right panel) and ability to form colonies both under adherent (Fig. 5b, right panel) and non-adherent (Fig. 5c, right panel) conditions. These increased abilities to grow and form colonies were not observed in H1703 T-RexB cells treated with doxycycline (Fig. 5a, b and c, middle panels), which do not express oncogenic KRAS. Interestingly, treatment with AI II abrogated the enhanced growth (Fig. 5a, right panel), colony formation (Fig. 5b, right panel) and anchorage-independent growth (Fig. 5c, right panel) of H1703 G12V cells induced to express KRAS^G12V^ with doxycycline, but had no effect in uninduced H1703 G12V cells (Fig. 5a, b and c, right panels) or in H1703 T-RexB cells (Fig. 5a, b and c, middle panels). Again, loss of viability was confirmed by MTT reduction-based assays, whereby MTT reduction is decreased upon treatment with AI II only in cells expressing mutant KRAS^G12V^ (Additional file 4: Figure S3A and B). Interestingly, as assessed by caspase 3 cleavage (Additional file 4: Figure S3C), or by Annexin V/PI staining (Additional file 4: Figure S3D), aurora kinase targeting with AI II preferentially increases apoptosis of KRAS^G12V^-expressing H1703 cells.

These results show that Aurora inhibition primarily exerts anti-tumor effects in the presence of oncogenic KRAS^G12V^, having little or no effect on normal cells or tumorigenic cells without KRAS mutations. This suggests that Aurora kinase inhibition therapy can preferentially target KRAS^G12V^ transformed cells.

## Discussion

In spite of recent studies that provide new promising strategies for targeting KRAS directly [1–4], effective therapies for KRAS-induced malignancies, including KRAS-induced lung cancer, are still unavailable. Targeting KRAS indirectly through its downstream effectors is also complicated by the number and crosstalk of pathways regulated by KRAS that contribute to the malignant phenotype [46, 47]. Effective indirect KRAS targeting is likely to rely on combined inhibition of critical targets, which may vary according to context. Therefore, a better understanding of the molecular pathways triggered by oncogenic KRAS in lung cancer is warranted, as it can identify new potential targets to be explored therapeutically.

In this work we demonstrated that AURKA and AURKB are potential new promising targets for KRAS-induced lung cancer therapy. Using three different cell-based models, we have shown that oncogenic KRAS positively regulates expression of AURKA and AURKB likely by regulating AURKA and AURKB transcription or mRNA stability (Fig. 1 and Additional file 2: Figure S1). An increase in Aurora A expression was also observed in a mouse model of malignant peripheral nerve sheath tumor caused by overexpression of HRAS^G12V^ [37]. These results are consistent with the fact that RAS activation leads to phosphorylation and stabilization of the MYC transcription factor [48], which in turn binds to the AURKA and AURKB promoters and positively regulates their transcription [49]. These results are also supported by the observation that AURKA and AURKB are required for in vivo maintenance of MYC-induced tumors [49, 50].

Nevertheless, we cannot rule out that other mechanisms, both transcriptional and post-transcriptional, may be involved in the regulation of KRAS-induced AURKA and AURKB activity. Recently, Bowman et al. [51] reported that KRAS can induce phosphorylation of FADD by CK1α, and that phosphorylated FADD is, not only required for KRAS-induced lung tumorigenesis, but also that it interacts with AURKA. In addition, Yang et al. [28] demonstrated that, in ovarian cancer cells, oncogenic HRAS led to accumulation of AURKA protein possibly via inhibition of AURKA proteolysis. Interestingly, AURKA and AURKB expression levels have been used as diagnostic and prognostic markers in lung cancer [16–19, 52], suggesting that these kinases play a role in lung oncogenesis.

In order to investigate if AURKA and AURKB contribute to the malignant phenotype induced by KRAS activation, we either targeted AURKA or AURKB expression by RNA interference or targeted their activity with a dual pharmacological inhibitor. By these approaches, we have shown that these kinases are important for growth and viability of KRAS-positive lung cancer cells (Figs. 2b–c and 3b–c). These results are consistent with other studies showing that these kinases are important for cancer cell growth, viability and in vivo tumorigenicity [37, 53–56]. Nonetheless, our findings demonstrate that these kinases also support the malignant phenotype of KRAS-transformed lung cancer cells.

We have determined that Aurora kinase targeting in KRAS-positive lung cancer cells leads to loss of viability through promoting cell cycle arrest at G2 and through activation of apoptosis (Figs. 2d–e and 3d–e). This is consistent with the fact that AURKA and AURKB are master regulators of mitosis [10, 11], and that Aurora kinase inhibition is known to promote cell cycle arrest and apoptosis [14, 15].

More importantly, Aurora kinase targeting reduced anchorage-independent growth of KRAS-transformed lung cells (Figs. 2f, 3f and 5c), and inducible inhibition of AURKA expression by RNA interference reduced A549 xenograft tumor growth in vivo (Fig. 4). Different studies have shown that Aurora targeting reduces cell transformation in vitro and tumorigenicity in vivo [50, 53–63], even though these studies did not evaluate Aurora kinase targeting in KRAS-driven oncogenesis. Different mechanisms have been proposed to explain the role these kinases play in promoting malignant transformation. One possible mechanism would be through phosphorylation of LIMK2 by AURKA [64]. Another possibility is that AURKA can promote RAS farnesylation, thus increasing the ability of this oncoprotein to transmit signals to downstream effectors [28]. In the case of AURKB, phosphorylation of CDCA8 by AURKB can play a role in promoting cell growth, survival and xenograft tumor growth [25]. Finally, both kinases have been proposed to mediate the oncogenic effects of MYC [49], an oncogenic transcription factor known to be positively regulated by RAS [48].

Interestingly, whereas AURKA targeting in vitro caused cells to undergo apoptosis (Figs. 2d and 3e and Additional file 3: Figure S2B, Additional file 4: Figure S3C and D), we failed to detect apoptotic cells in xenograft tumors with RNAi-mediated inihibition of AURKA expression (data not shown). This discrepancy between our in vitro and in vivo data could reflect the fact that tumor extrinsic survival factors influenced by the microenvironment in vivo can affect the ability of tumors cells to undergo cell death upon inhibition of oncogenic pathways. It is known that several key stages in apoptosis are sensitive to depletion of cellular energy reserves, which results from conditions, such as hypoxia and low glucose, which are found in vivo [65]. In addition, hypoxia is known to select cells that have lost their apoptotic potential [66], so it is also likely that an initial apoptotic response to AURKA inhibition might have occurred, leading to expansion of apoptotic resistant cells and failure to detect apoptotic cells at the endpoint.

In order to validate Aurora kinases as therapeutic targets for KRAS-induced lung cancer, it is important to show that inhibiting these kinases preferentially targets lung cancer cells expressing oncogenic KRAS. In order to evaluate the specificity of the biological effects caused by Aurora kinase targeting, we used the dual AURKA and AURKB inhibitor AI II to treat lung cell pairs, that only differed by their expression of oncogenic KRAS^G12V^. Our results show, for the first time, that AURKA and AURKB inhibition reduces growth, viability and anchorage- independent growth in a KRAS-dependent manner (Fig. 5 and Additional file 4: Figure S3).

Our results support a model, whereby oncogenic KRAS effector pathways trigger abnormally high levels of AURKA and AURKB activation, rendering KRAS-transformed cells more sensitive than other cells to Aurora kinase targeting. These results are important, not only because they identify a specific vulnerability of KRAS-transformed lung cells, but also because this vulnerability can be used to develop targeted therapies for KRAS-induced lung cancer. The potential for clinical translation is further underscored by the fact that different Aurora kinase inhibitors, including alisertib and danusertib are undergoing phase II/III clinical trials for different malignancies [15, 59, 67, 68], even though very few combination trials using these drugs are currently ongoing.

Taken together, our results provide strong evidence that Aurora kinases are KRAS downstream effectors that play an important role in promoting the oncogenic phenotype, and suggest AURKA and/or AURKB inhibition as a promising approach to be explored alone or preferentially in combination with other strategies for KRAS-induced lung cancer therapy.

## Conclusions

In conclusion, our results show that AURKA and AURKB are not only regulated by KRAS, but also promote KRAS-induced oncogenesis. Furthermore, targeting Aurora kinases by RNA interference reduces the oncogenic phenotype of KRAS mutant lung cancer cells in vitro and AURKA targeting slows tumor growth in vivo. Finally, Aurora kinase pharmacological inhibition preferentially targets lung cancer cells expressing KRAS^G12V^, thereby supporting our hypothesis that AURKA and AURKB are promising targets for KRAS-induced lung cancer therapy.

## Methods

### Cell lines, antibodies and reagents

A549 (ATCC CCL-185) and H358 (ATCC CRL-5807) cells were cultivated in RPMI-1640 medium (ATCC 30–2001) supplemented with 10 % (vol/vol) fetal bovine serum (FBS), without antibiotics. HEK 293 T/17 cells (ATCC CRL-11268) were cultivated in Dulbecco’s Modified Eagle’s Medium (DMEM) supplemented with 10 % (vol/vol) FBS, without antibiotics. H1703 (ATCC CRL-5889) genetically modified to inducibly express KRAS^G12V^ or empty vector control (T-RexB) [40] were cultivated in RPMI-1640 medium supplemented with 10 % (vol/vol) FBS, 10 μg/mL blasticidin (#A1113903, Invitrogen) and 3 μg/mL zeocin (#R25001, Invitrogen). 2 μg/mL doxycycline (#D3447, Sigma Aldrich) was added to the medium to induce KRAS^G12V^ expression when appropriate (see figure legends). SALEB and SAKRAS cells [39] were a kind gift from Dr. Scott Randell, UNC School of Medicine. They were cultured in supplemented Bronchial Epithelial Cell Growth Medium (BEGM, Lonza). The antibodies used were as follows: Anti-AURKA (#4718, Cell Signaling Technology), anti-pAURKA (#3079, Cell Signaling Technology), anti-AURKB (#3094, Cell Signaling Technology), anti-GAPDH (#25778, Santa Cruz Biotechnology), anti-HA-tag (#H3663, Sigma-Aldrich), anti-PanRAS (#OP40, Merck Millipore), anti-cleaved caspase 3 (#9661, Cell Signalling), anti-full caspase 3 (#9662, Cell Signalling), anti-β-actin (#7210, Santa Cruz Biotechnology) and anti-α-tubulin (#T9026, Sigma-Aldrich). Secondary antibodies were as follows: anti-rabbit Alexa Fluor® 680 (#A-21109 Life Technologies), anti-mouse Alexa Fluor® 680 (#A-21058 Life Technologies), anti-rabbit IRDye® 800CW (#926-32211, Li-Cor) and anti-mouse IRDye® 800CW (#926-32210, Li-Cor). Cells were treated as described in figures with Aurora Inhibitor II (AI II, #189404, Merck Millipore), which is a dual Aurora A and Aurora B inhibitor.

### Generation of lung cells with inducible inhibition of KRAS, Aurora A or Aurora B expression by RNA interference

Lentiviral particles were produced in HEK 293 T/17 cells by co-transfection of lentiviral packaging plasmids pCMV-VSVG and pCMV-dR8.2 dvpr (Addgene) with pTRIPZ lentiviral vectors (GE Dharmacon) expressing the desired shRNAs according to the manufacturer’s protocol. The following pTRIPZ lentiviral vectors were used: a lentiviral vector expressing a non-targeting control shRNA (shCtrl, RHS 4743), two lentiviral vectors expressing different shRNAs targeting KRAS (shKR#1, V3THS_314004 and shKR#2, V3THS_314009), two lentiviral vectors expressing different shRNAs targeting Aurora A (shAKA#1, V2THS_12364 and shAKA#2, V2THS_153609) and two lentiviral vectors expressing different shRNAs targeting Aurora B (shAKB#1, V2THS_28606 and shAKB#2, V2THS_28601). In each case, A549 and H358 cells were infected with 1 MOI of lentiviral particles and selected with 2 μg/mL puromycin (#A1113803, Life Technologies) for two weeks. Individual cell clones were induced with 2 μg/mL doxycycline (#D9891, Sigma-Aldrich) for 5 days and screened for expression of the reporter red fluorescent protein (turboRFP), as well as for inhibition of expression of shRNA targets. Cells with at least 80 % knockdown levels were used to perform all biological assays.

### Growth curve analysis

Cells were seeded at a confluence of 5 × 10^3^ cells per well in 6-well adherent plates and allowed to proliferate for 12 days with medium changed daily. Cells were counted every two 2 days. Trypan blue (#T8154, Sigma-Aldrich) was used to exclude dead cells. All conditions were done in triplicate.

### Clonogenic assay

Cells were seeded at 500 cells per plate in 60 mm adherent plates in triplicate. Cells were allowed to form colonies for 2 weeks with medium changed daily. Colonies were stained with crystal violet solution and counted manually.

### Soft agar assay

1 × 10^3^ cells were ressuspended in cell culture medium containing 0.3 % low-melting-point agarose and were plated onto a solidified bottom layer medium containing 0.6 % agarose in 24-well plates. Liquid medium was added after 24 h and changed every three days. Colonies were allowed to grow for 3 weeks and subsequently stained with 1 mg/mL 3-(4,5-dimethylthiazol-2-yl)-2,5-diphenyltetrazolium bromide (MTT, #M5655, Sigma-Aldrich) for 2 h and colonies were counted. All conditions were done in sextuplicate.

### Western blotting

1 × 10^5^ cells were grown in 60 mm plates and whole cell lysates were isolated using RIPA buffer (20 mM Tris–HCl pH 7.5, 150 mM NaCl, 1 mM Na_2_EDTA, 1 mM EGTA, 1 % NP-40) supplemented with protease and phosphatase inhibitors (1 μg/mL leupeptin, 1 % sodium deoxycholate, 2.5 mM sodium pyrophosphate, 1 mM b-glycerophosphate, 1 mM Na^3^VO_4_). 25 μg of protein/lane were electrophoresed in 10 or 12 % polyacrylamide minigels at 120 V for 90 min and transferred to nitrocellulose membranes. After blocking, the membranes were incubated with primary antibody, followed by incubation with fluorescently labelled secondary antibodies. Fluorescence detection was performed using ODYSSEY® CLx (Li-Cor®).

### AURKA phosphorylation assay

1 × 10^5^ cells were grown in 60 mm plates and treated with Aurora inhibitor II (#189404, Merck Millipore) for 72 h. 24 h after cell seeding, 2 mM thymidine (#T1895, Sigma- Aldrich) was added. The thymidine block was released after 24 h of incubation and cells were cultured for an additional 8 h. Finally, 100 ng/mL nocodazole (#M1404, Sigma-Aldrich) was added for 16 h. Cells were harvested and whole lysates were analyzed by Western blotting to check for AURKA phosphorylation.

### Cell cycle analysis

Cells were seeded at a confluence of 1 × 10^5^ cells in 6-well adherent plates. After drug treatment or doxycycline-mediated expression of shRNAs, cells were incubated with 50 μM 5-Bromo-2′-deoxyuridine (BrdU, #19-160, Merck Millipore) in the dark for 2 h, and fixed in 70 % ethanol. Cells were treated with 100 μg/mL RNase A (#EN0531, Thermo Fisher Scientific) for 30 min at 37 °C followed by 2 M HCl for 20 min at room temperature. Suspended cells were incubated in 100 μL of Alexa Fluor® 488-labeled BrdU antibody (#FCMAB101A4, Merck Millipore) for 1 h in the dark, and finally resuspended in a buffer containing 100 μg/mL of propidium iodide (#81845, Sigma-Aldrich) for analysis using flow cytometry in a BD FACSVerse™ Flow Cytometer (BD Biosciences). Results were analyzed with Kaluza® Flow Analysis Software (Beckman Coulter).

### Annexin V/PI staining

Cells were seeded at a confluence of 1 × 10^6^ cells in 100 mm adherent plates and treated with different concentrations of AI-II or doxycycline as indicated in the figure legends. Cells were stained with Annexin V and propidium iodide (PI) according to the Annexin V-FITC Kit (#130-092-052, Miltenyi Biotec) instructions. Briefly, cells were resuspended in binding buffer, incubated with FITC-labeled Annexin V antibody for 15 min, followed by incubation in binding buffer containing 100 μg/mL propidium iodide (PI). Cells were then analyzed by flow cytometry in a BD FACSVerse™ Flow Cytometer (BD Biosciences). Results were analyzed with Kaluza® Flow Analysis Software (Beckman Coulter).

### Xenograft tumor growth analysis

2 × 10^6^ A549 cells with doxycycline-inducible expression of a shRNA targeting AURKA (A549-shAKA) or with doxycycline-inducible expression of a non-targeting shRNA (A549-shCtrl) were subcutaneously inoculated into the flanks of 8-week-old, male Balb/C nude mice in pathogen-free conditions according to the protocols approved by the Institutional Animal Care and Use Committee of the University of São Paulo Chemistry Institute Animal Facility. Animals were monitored every 3 days, and, once primary tumors were palpable (26 days after inoculation), 1 mg/mL doxycycline was added to the drinking water to induce shRNA expression. Tumor volumes were determined by direct diameter measurement with calipers followed by the following calculation: (large diameter) x (small diameter)^2^/2. Animals were sacrificed by CO_2_ euthanasia 56 days after inoculation, and tumor weight was determined. Statistical significance of differences observed in tumor growth and weight was determined by one-way analysis of variance (ANOVA) followed by Bonferroni’s multiple comparison test or by the Student's *t*-test.

### Statistics

All values are presented either as mean ± 1SD or as representative images of at least three independent experiments. Data analysis was performed using the statistical software Prism 5 (GraphPad Software, Inc., San Diego, CA, USA). Differences between groups were evaluated by one-way analysis of variance (ANOVA) followed by Bonferroni’s multiple comparison test or alternatively by the Student's *t*-test. Differences were considered statistically significant at *p* < 0.05.

## Additional files

Additional file 1:
**Materials and methods used in the data presented in the additional figures, which are not included in the main manuscript.** (PDF 80 kb) Additional file 2: Figure S1. KRAS regulates AURKA and AURKB mRNA levels. **a** Expression of *KRAS, AURKA,* and *AURKB* was analyzed by real-time quantitative PCR in immortalized primary epithelial lung cells (SALEB) and their isogenic KRAS-transformed counterpart (SAKRAS), showing that KRAS mRNA expression positively correlates with both AURKA and AURKB mRNA expression. Statistical significance was measured by Student’s *t*-test (**p* < 0.05) and error bars represent average ± 1s.d. **b** mRNA expression of *KRAS, AURKA,* and *AURKB* was analyzed by real-time quantitative PCR in H1703 lung cancer cells engineered to express KRAS^G12V^ inducibly (G12V) in comparison to empty vector-transfected H1703 cells (TrexB). To induce KRAS expression, cells were treated with 2 μg/mL doxycycline for the indicated times. Statistical significance was measured by Student’s *t*-test (**p* < 0.05) by comparing treated samples (DOX) to untreated control samples (MOCK). Error bars represent average ± 1s.d. **c** Fluorescence microscopy images of A549 and H358 stable cells with inducible expression of 2 different shRNAs targeting KRAS (shKR#1 and shKR#2), AURKA (shAKA#1 and shAKA#2), AURKB (shAKB#1 and shAKB#2) or a non-targeting shRNA (shCtrl) showing induction of red fluorescent reporter protein (RFP) expression upon treatment with 2 μg/mL doxycycline for 5 days (DOX) when compared to untreated (MOCK) cells. Brightfield images of the same fields are included for comparison. **d** mRNA expression of *KRAS, AURKA,* and *AURKB* was analyzed by real-time quantitative PCR in A549 and H358 stable cells with inducible expression of 2 different shRNAs targeting KRAS (shKR#1 and shKR#2) or a non-targeting shRNA (shCtrl). Cells were either treated with 2 μg/mL doxycycline (DOX) for 5 days to induce shRNA expression or left untreated (MOCK). Statistical significance was measured by Student’s *t*-test (**p* < 0.05, ***p* < 0.01) by comparing treated samples (DOX) to untreated control samples (MOCK). Error bars represent average ± 1s.d. (PDF 648 kb) Additional file 3: Figure S2. Pharmacological or shRNA-mediated inhibition of AURKA and/or AURKB leads to decreased cell viability and induces apoptosis. **a** A549 and H358 cells were treated with 0.1 % DMSO or 1uM AI II for 72h. Cell viability was measured using a colorimetric MTT assay (described in the Additional file 1: Methods). **b** Protein lysates of A549 and H358 cells treated with 0.1 % DMSO or the indicated concentrations of AI II for 72h were submitted to western blotting with the indicated antibodies. C3) anti-caspase 3; ACTB) anti-β-actin. **c** A549 and H358 stable cells with inducible expression of 2 different shRNAs targeting AURKA (shAKA#1 and #2), AURKB (shAKB#1 and #2) or a non-targeting shRNA (shCtrl) were treated with 2 μg/mL doxycycline (DOX) for 5 days to induce shRNA expression. Cell viability of doxycycline-treated cells was compared to untreated cells using a colorimetric MTT assay (described in the Additional file 1: Methods). Statistical significance in all cases was measured by Student’s *t*-test (***p* < 0.01, ****p* < 0.0001) when compared to experimental control samples (MOCK-treated cells). Error bars represent average ± 1s.d. (PDF 263 kb) Additional file 4: Figure S3. Aurora inhibition reduces viability and induces cell death in a KRAS-dependent manner. **a** Primary immortalized human airway cells (SALEB) and their KRAS-transformed counterpart (SAKRAS) were treated with 0.1 % DMSO or 1uM AI II. Cell viability was measured at 72h using a colorimetric MTT assay (described in the Additional file 1: Methods). Statistical significance was determined by Student’s *t*-test (**p* < 0.05) when compared to experimental control samples (DMSO-treated cells). Error bars represent average ± 1s.d. **b**, **c** and **d** H1703-TrexB and H1703-G12V lung cancer cells were treated with 0.1 % DMSO (DMSO) or 1μM AI II (AI II) as indicated for 72h. To induce KRAS expression H1703-TrexB and H1703-G12V cells were simultaneously treated with 2μg/mL doxycycline (DOX + DMSO or DOX + AI II) where indicated. **b** Cell viability was measured using a colorimetric MTT assay (described in the Additional file 1: Methods). Statistical significance was determined by Student’s *t*-test (**p* < 0.05, ****p* < 0.0001) when compared to experimental control samples (DMSO-treated cells). Error bars represent average ± 1s.d. **c** Protein lysates were submitted to western blotting with the indicated antibodies. C3) anti-caspase 3. **d** Annexin V positive cells were analyzed by flow cytometry. (PDF 455 kb)
