# Correction: Aurora kinase targeting in lung cancer reduces KRAS-induced transformation

**DOI:** 10.1186/s12943-024-01964-6

**Published:** 2024-03-09

**Authors:** Edmilson Ozorio dos Santos, Tatiana Correa Carneiro-Lobo, Mateus Nobrega Aoki, Elena Levantini, Daniela Sanchez Bassères

**Affiliations:** 1 Department of Biochemistry, Chemistry Institute, University of São Paulo, São Paulo, SP Brazil; 2 Beth Israel Deaconess Medical Center, Harvard Medical School, Boston, MA USA; 3 Institute of Biomedical Technologies, National Research Council (CNR), Pisa, Italy

**Correction: Mol Cancer 15, 12 (2016)**

**https://doi.org/10.1186/s12943-016-0494-6**

Following publication of the original article [1], the authors identified an error in Fig. 1f. The correct and incorrect figures are given below.

Incorrect Fig. 1:
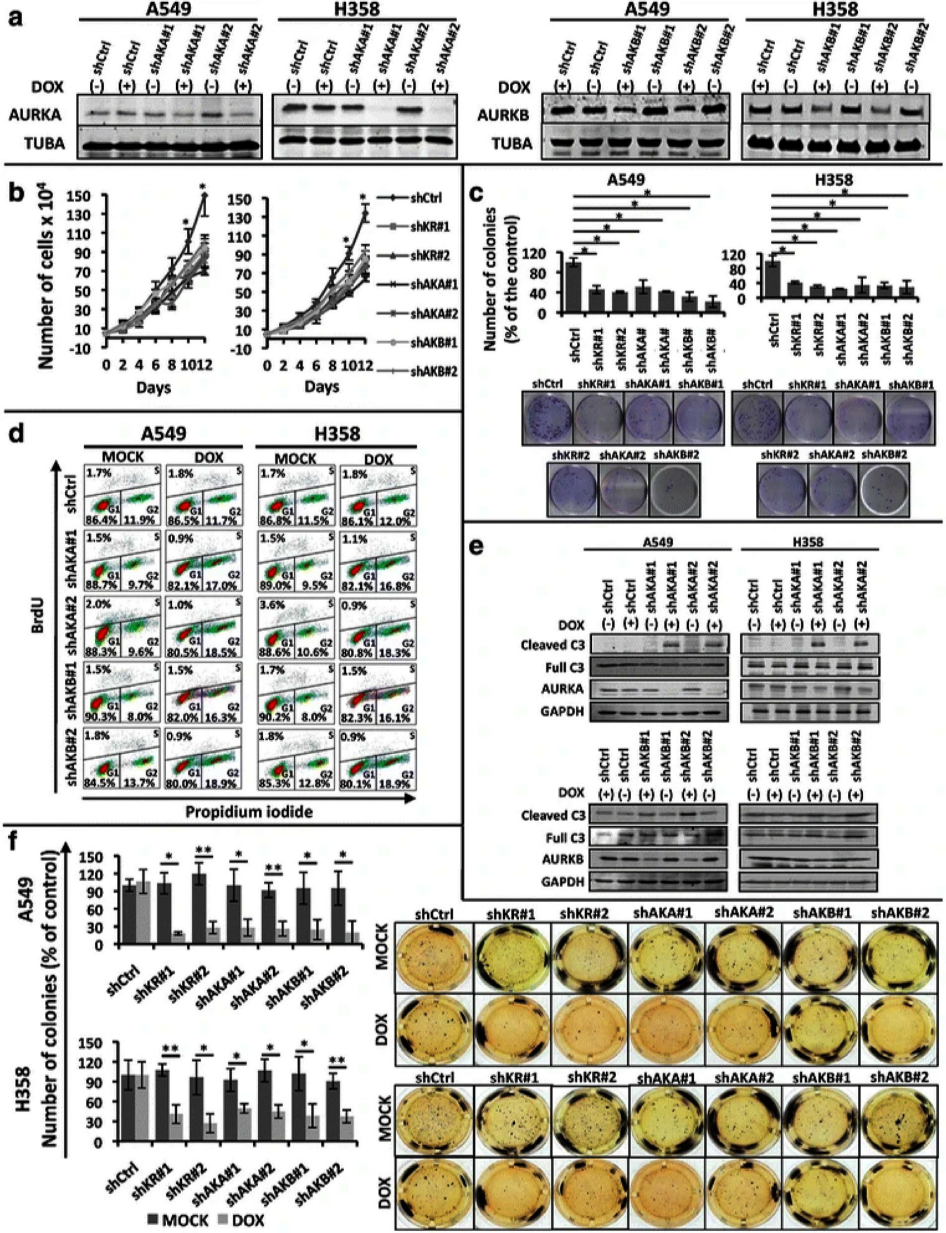


Correct Fig. 1:

**Fig. 1:**
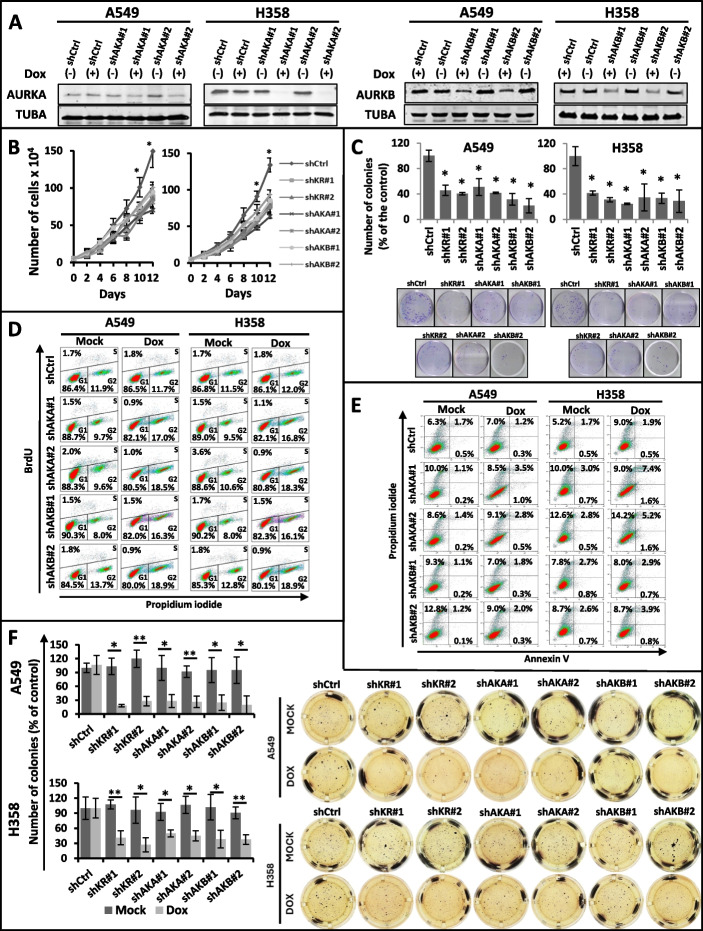
shRNA-mediated knockdown of AURKA or AURKB decreases the transformed phenotype of KRAS-positive lung cells. Unless otherwise indicated, A549 and H358 stable cells with inducible expression of 2 different shRNAs targeting AURKA (shAKA#1 and shAKA#2), AURKB (shAKB#1 and shAKB#2) or a non-targeting shRNA (shCtrl) were either treated with 2 μg/mL doxycycline (DOX) for 5 days to induce shRNA expression or left untreated (MOCK). **a** Protein lysates of doxycycline-treated ( +) and untreated ( −) cells were submitted to western blotting with the indicated antibodies. TUBA) anti-α-tubulin. **b** Growth curve analysis of the indicated cells. All cells were treated with 2 μg/mL doxycycline (DOX) for the indicated times. **c** The indicated cells were plated for clonogenic assays as described in methods and treated for 21 days with 2 μg/mL doxycycline (DOX). Colonies formed were stained with crystal violet and counted. Images shown are representative of three independent experiments. **d** The indicated treated (DOX) or untreated (MOCK) cells were stained with BrdU and propidium iodide (PI) as described in methods, and cell cycle analysis was performed by flow cytometry. **e** Protein lysates of A549 and H358 stable cells with inducible expression of 2 different shRNAs targeting AURKA (shAKA#1 and #2), AURKB (shAKB#1 and #2) or a non-targeting shRNA (shCtrl), treated ( +) or not (-) with 2 μg/mL doxycycline (DOX) for 5 days, were submitted to western blotting with the indicated antibodies. C3) anti-caspase 3. **f** Anchorage-independent growth was evaluated by plating the indicated cells in soft agar as described in methods. Cells were then treated for 21 days with 2 μg/mL doxycycline (DOX) or left untreated (MOCK). Colonies formed were stained with MTT and counted. Images shown are representative of three independent experiments. In all cases, statistical significance was determined when appropriate by Student’s *t*-test (*p < 0.05, **p < 0.01) and the groups being compared are indicated by horizontal bars.
