# Supplementary material for: Aurora kinase targeting in lung cancer reduces KRAS-induced transformation
Source: Mol Cancer. 2016 Feb 3;15:12. doi: 10.1186/s12943-016-0494-6 (PMC4739397; doi:10.1186/s12943-016-0494-6)
Supplement: Additional file 1: — Materials and methods used in the data presented in the additional figures, which are not included in the main manuscript. (PDF 80 kb) [file 12943_2016_494_MOESM1_ESM.pdf]

Additional Material and Methods

cDNA synthesis and RT-qPCR. Total RNA was extacted using TriReagent solution (#AM9738, Life Technologies) according to the manufacturer’s instructions. 1ug RNA was used to synthesize cDNA using M-MLV reverse transcriptase (#M1701, Promega), and real-time PCR analysis was performed with ABI PRISM® 7900HT Sequence Detection System (#4317596, Applied Biosystems®) using SYBR® Green master mix (#4472908, Life Technologies) and gene-specific forward or reverse primers. The sequences and concentrations used for each primer pair were as follows: AURKA (200 nM) Seq_{Fw}: GAAAGCCGGAGTGGAGCAT, Seq_{Rv}: TGCCGAAGGTGGGACTGTAT; AURKB (200 nM) Seq_{Fw}: TGTCACCCCATCTGCACTTG, Seq_{Rv}: CAGCTGTGGGCTGGACATT; KRAS (200 nM) Seq_{Fw}: CCCAGGTGCGGGAGAGA; Seq_{Rv}: CAGCTCCAACTACCACAAGTTT; GAPDH (800 nM) Seq_{Fw}: GAGCCGCATCTTCTTTTGC, Seq_{Rv}: CCATGGTGTCTGAGCGATGT; GUSB (200 nM) Seq_{Fw}: CTCATTTGGAATTTTGCCGATT, Seq_{Rv}: CCGAGTGAAGATCCCCTTTTT; ACTB (400 nM) Seq_{Fw}: GGCACCCAGCACAATGAAG, Seq_{Rv}: CCGATCCACACGGGAGTACTTG. Relative quantitation was determined by the $\Delta\Delta C_t$ method using GUSB, GAPDH or ACTB as endogenous controls.

3-(4,5-dimethylthiazol-2-yl)-2,5-diphenyltetrazolium bromide- (MTT)-reduction based viability assay. 1 x 10^3 cells were seeded in 96-well adherent plates. After drug treatment, cells were incubated with 1 mg/mL MTT (#M5655, Sigma-Aldrich) for 2 hours and formazan crystals were then resuspended in DMSO. The reduction of MTT

to formazan was measured colorimetrically at 570 nm in a EON™ plate reader (Biotek®). All conditions were done in octuplicate.
