# Supplementary figures and images for: Aurora kinase targeting in lung cancer reduces KRAS-induced transformation
Source: Mol Cancer. 2016 Feb 3;15:12. doi: 10.1186/s12943-016-0494-6 (PMC4739397; doi:10.1186/s12943-016-0494-6)

# FIGURE S1

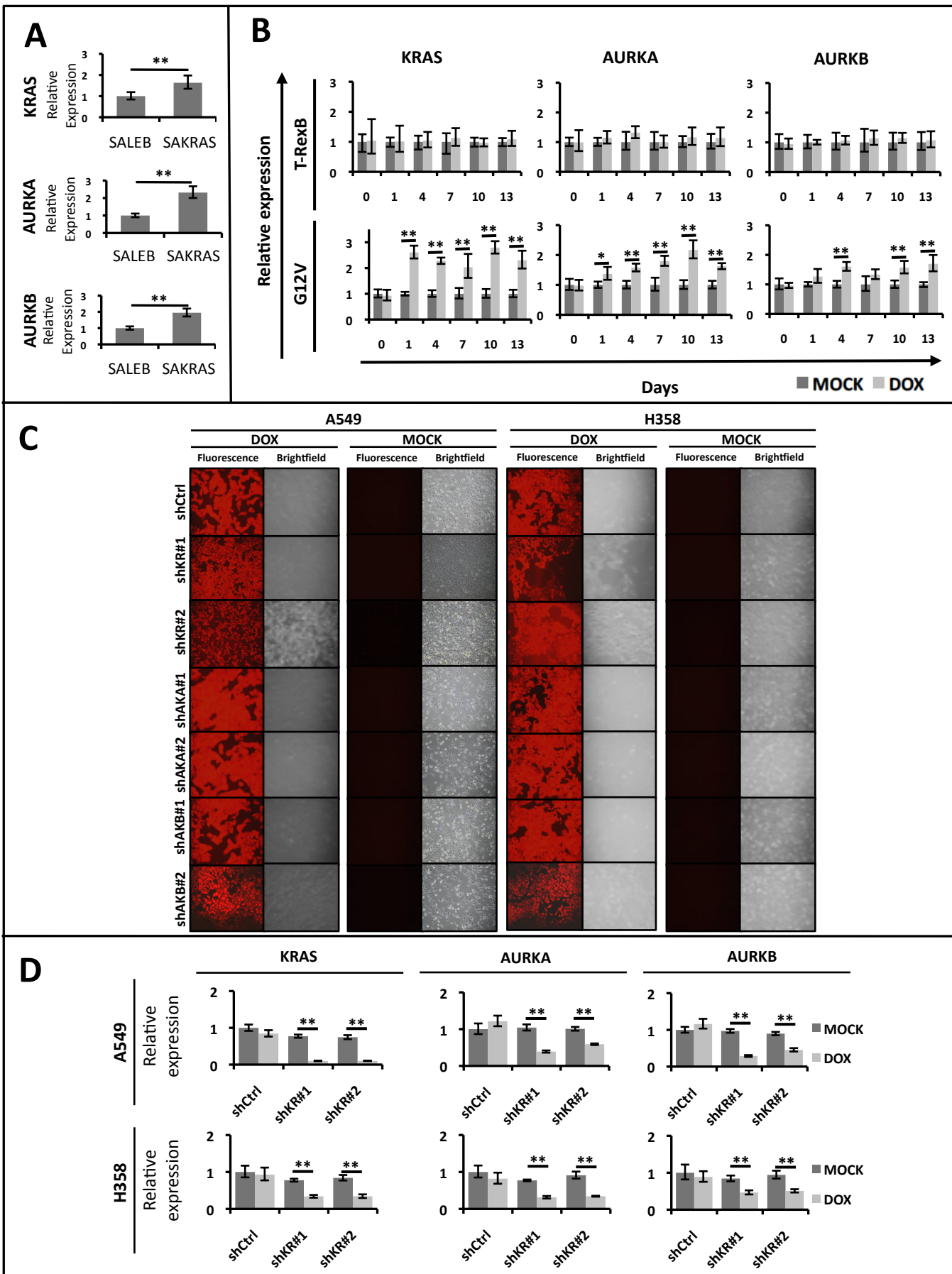

Supplement: Additional file 2: Figure S1. — KRAS regulates AURKA and AURKB mRNA levels. a Expression of KRAS, AURKA, and AURKB was analyzed by real-time quantitative PCR in immortalized primary epithelial lung cells (SALEB) and their isogenic KRAS-transformed counterpart (SAKRAS), showing that KRAS mRNA expression positively correlates with both AURKA and AURKB mRNA expression. Statistical significance was measured by Student’s t-test (*p < 0.05) and error bars represent average ± 1s.d. b mRNA expression of KRAS, AURKA, and AURKB was analyzed by real-time quantitative PCR in H1703 lung cancer cells engineered to express KRASG12V inducibly (G12V) in comparison to empty vector-transfected H1703 cells (TrexB). To induce KRAS expression, cells were treated with 2 μg/mL doxycycline for the indicated times. Statistical significance was measured by Student’s t-test (*p < 0.05) by comparing treated samples (DOX) to untreated control samples (MOCK). Error bars represent average ± 1s.d. c Fluorescence microscopy images of A549 and H358 stable cells with inducible expression of 2 different shRNAs targeting KRAS (shKR#1 and shKR#2), AURKA (shAKA#1 and shAKA#2), AURKB (shAKB#1 and shAKB#2) or a non-targeting shRNA (shCtrl) showing induction of red fluorescent reporter protein (RFP) expression upon treatment with 2 μg/mL doxycycline for 5 days (DOX) when compared to untreated (MOCK) cells. Brightfield images of the same fields are included for comparison. d mRNA expression of KRAS, AURKA, and AURKB was analyzed by real-time quantitative PCR in A549 and H358 stable cells with inducible expression of 2 different shRNAs targeting KRAS (shKR#1 and shKR#2) or a non-targeting shRNA (shCtrl). Cells were either treated with 2 μg/mL doxycycline (DOX) for 5 days to induce shRNA expression or left untreated (MOCK). Statistical significance was measured by Student’s t-test (*p < 0.05, **p < 0.01) by comparing treated samples (DOX) to untreated control samples (MOCK). Error bars represent average ± 1s.d. (PDF 648 kb) [file 12943_2016_494_MOESM2_ESM.pdf]

# FIGURE S2

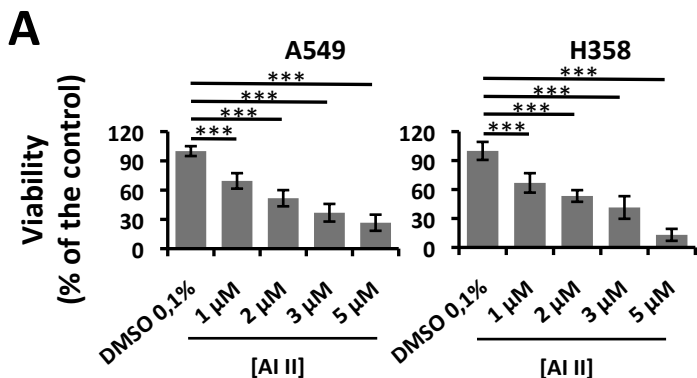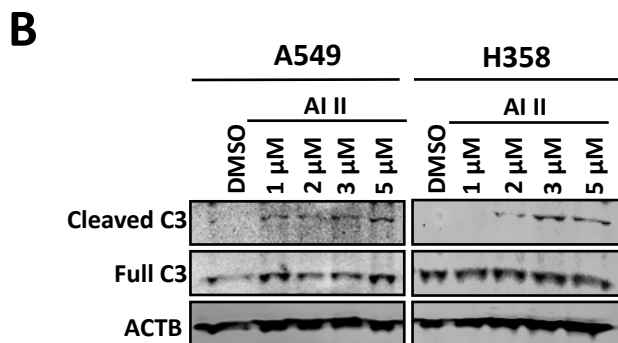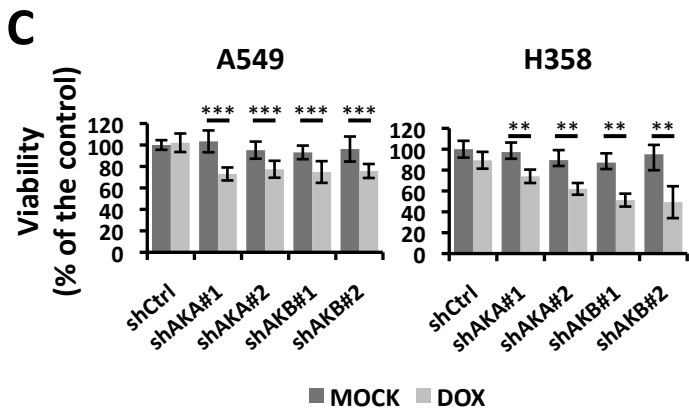

Supplement: Additional file 3: Figure S2. — Pharmacological or shRNA-mediated inhibition of AURKA and/or AURKB leads to decreased cell viability and induces apoptosis. a A549 and H358 cells were treated with 0.1 % DMSO or 1uM AI II for 72h. Cell viability was measured using a colorimetric MTT assay (described in the Additional file 1: Methods). b Protein lysates of A549 and H358 cells treated with 0.1 % DMSO or the indicated concentrations of AI II for 72h were submitted to western blotting with the indicated antibodies. C3) anti-caspase 3; ACTB) anti-β-actin. c A549 and H358 stable cells with inducible expression of 2 different shRNAs targeting AURKA (shAKA#1 and #2), AURKB (shAKB#1 and #2) or a non-targeting shRNA (shCtrl) were treated with 2 μg/mL doxycycline (DOX) for 5 days to induce shRNA expression. Cell viability of doxycycline-treated cells was compared to untreated cells using a colorimetric MTT assay (described in the Additional file 1: Methods). Statistical significance in all cases was measured by Student’s t-test (**p < 0.01, ***p < 0.0001) when compared to experimental control samples (MOCK-treated cells). Error bars represent average ± 1s.d. (PDF 263 kb) [file 12943_2016_494_MOESM3_ESM.pdf]

FIGURE S3

**A**

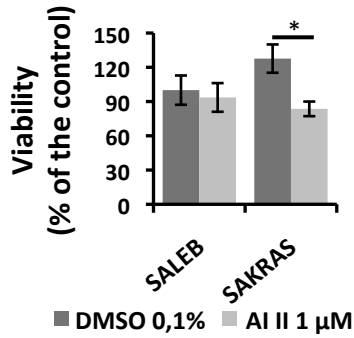

**B**

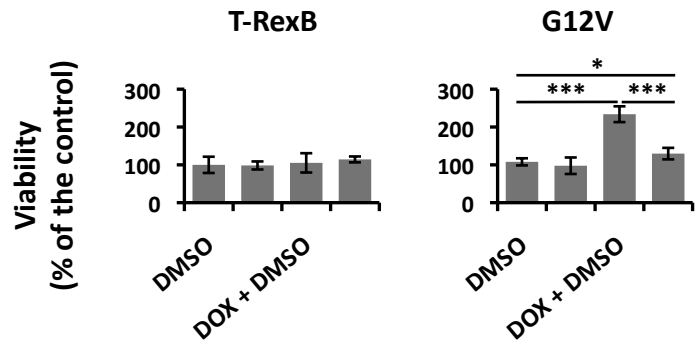

**C**

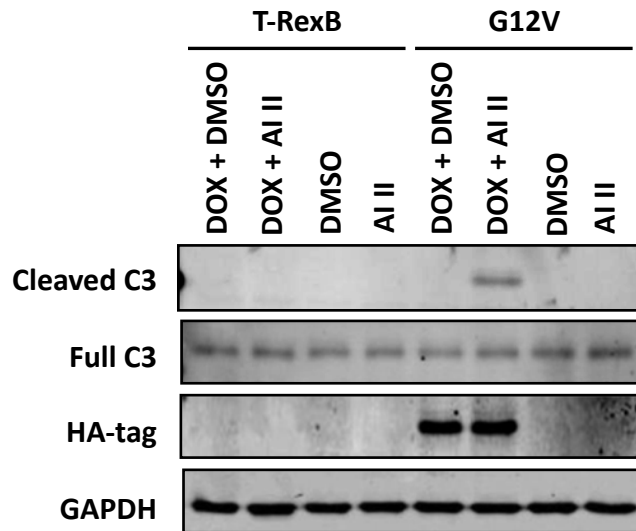

**D**

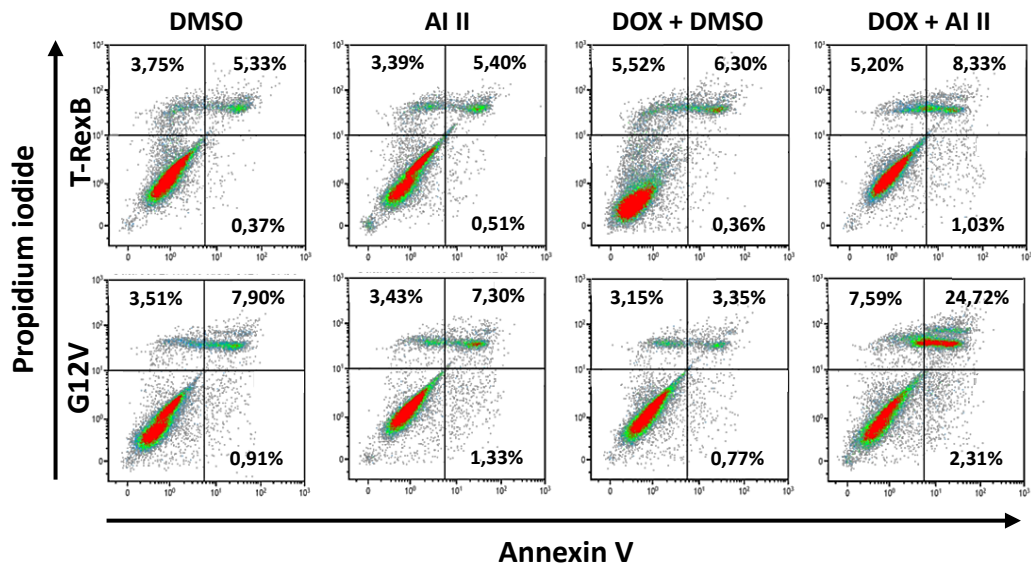

Supplement: Additional file 4: Figure S3. — Aurora inhibition reduces viability and induces cell death in a KRAS-dependent manner. a Primary immortalized human airway cells (SALEB) and their KRAS-transformed counterpart (SAKRAS) were treated with 0.1 % DMSO or 1uM AI II. Cell viability was measured at 72h using a colorimetric MTT assay (described in the Additional file 1: Methods). Statistical significance was determined by Student’s t-test (*p < 0.05) when compared to experimental control samples (DMSO-treated cells). Error bars represent average ± 1s.d. b, c and d H1703-TrexB and H1703-G12V lung cancer cells were treated with 0.1 % DMSO (DMSO) or 1μM AI II (AI II) as indicated for 72h. To induce KRAS expression H1703-TrexB and H1703-G12V cells were simultaneously treated with 2μg/mL doxycycline (DOX + DMSO or DOX + AI II) where indicated. b Cell viability was measured using a colorimetric MTT assay (described in the Additional file 1: Methods). Statistical significance was determined by Student’s t-test (*p < 0.05, ***p < 0.0001) when compared to experimental control samples (DMSO-treated cells). Error bars represent average ± 1s.d. c Protein lysates were submitted to western blotting with the indicated antibodies. C3) anti-caspase 3. d Annexin V positive cells were analyzed by flow cytometry. (PDF 455 kb) [file 12943_2016_494_MOESM4_ESM.pdf]
